# Supplementary material for: Establishment and validation of an interactive web-based calculator for predicting postoperative functional recovery in metatarsal fracture patients: A LASSO regression model approach
Source: PLoS One. 2025 Jun 3;20(6):e0323609. doi: 10.1371/journal.pone.0323609 (PMC12132955; doi:10.1371/journal.pone.0323609)
Supplement: Attachment — Submitted filename: Response to Reviewers.doc [file pone.0323609.s002.doc]

Responses to Questions

Dear Editors and Reviewers:

First of all, we sincerely thank you for taking the time to carefully review our paper. Your valuable comments and suggestions are of great significance in improving the quality of our manuscript. We have carefully studied each of your review comments and made targeted revisions and improvements accordingly.

Your professional insights have helped us identify the shortcomings in our paper, enabling us to re-examine our research work from a more rigorous and comprehensive perspective. These constructive comments not only helped improve the current paper but also provided important inspiration for our future research work.

We have made careful revisions based on your suggestions and hope that the revised version meets your requirements. If there are still any deficiencies, please continue to provide your guidance and criticism.

Thank you again for your hard work and valuable time!

Review Comments to the Author

Reviewer #1: Overall: This manuscript is written well and adds to the bulk of the literature
Abstract: Written well. Please, describe briefly postoperative functional recovery

Reply:Thank you very much for your valuable comment.The changes have been made as requested.(page 2,Abstract-result part,line 12-16.)

Methods:
- Please, identify the design of the study i.e. retrospective cohort

Reply:Thank you very much for your valuable comment.I apologize for my carelessness.The changes have been made as requested.(page 3,Method-Patient selection part,line 5.)

- When you mentioned "incomplete" clinical data, what do you mean by that? Any specific percentage for missing data based on which the participants would be excluded.

Reply:Thank you very much for your valuable comment.Incomplete clinical data refers to patients who had missing data related to preoperative factors, intraoperative factors, and postoperative factors(33 Predictive Factors) that we collected before follow-up, and these patients were excluded from the study.

(page 5,rusult-Study populations part,line 3-4,reference 34.)Generally speaking, a missing data rate below 10% of the total data is considered acceptable. In this study, the missing data rate was 5.17%.

- What is the reason for picking 2020 as the year for validation cohort?

Reply:Thank you very much for your valuable comment.1. Natural Time Division: The study period was from January 2018 to December 2022 (5 years). Using 2020 as the validation year creates a natural split with two years before and two years after in the training cohort, providing a balanced temporal distribution.

2. Sample Size Balance: This division resulted in a reasonable distribution of cases between training and validation cohorts, which is approximately a 3:1 ratio - a commonly used proportion in predictive modeling studies.

3. Using a complete calendar year (2020) as the validation cohort helps ensure temporal independence between the training and validation datasets, which is important for robust model validation.

- Data collection: It is very important to identify what postoperative functional recovery is. How it was evaluated? Subjective or objective assessment?

Reply:Thank you very much for your valuable comment..The changes have been made as requested.(page 4,Data collection part,paragraph2)

- The AO/OTA classification system is well established. However, it is still important to briefly describe it in the methods and cite proper citation for that.

Reply:Thank you very much for your valuable comment.The changes have been made as requested.(page 4,Data collection part,paragraph3,line 2-7,Reference 28)

- Statistical analysis: Did you evaluate the normality of data for continuous variables and how did you present them?
Reply:Thank you very much for your valuable comment.As all continuous variables (age, AOFAS midfoot functional score, and length of hospital stay) were categorized for analysis, normality testing was not necessary since the data were analyzed using categorical statistical methods.

Results:
- It is important to mention how many patients were included each year to see the representation

Reply:Thank you very much for your valuable comment.The changes have been made as requested.(page 6,Results-Study populations,paragraph1,line4-6.)

- It is important to mention how many patients had postoperative functional recovery as I do not see this well presented. This would also give us a clue if there is enough data to be included in the model and that there is no overfitting as per the rule of thumb.

Reply:Thank you very much for your valuable comment.The changes have been made as requested.(page 6,Results-Study populations part,paragraph1,line 8-10.)

- Consider building a prediction model for hospital stay as it worth exploring the factors that are associate with prolonged stay
Reply:Thank you very much for your valuable comment.While developing a predictive model for factors associated with prolonged hospital stay would be valuable, we decided to focus on descriptive and comparative analyses in this initial study. This decision was made for several reasons: First, predictive modeling requires a larger sample size to ensure model stability and prevent overfitting, particularly when dealing with multiple variables. Second, the complex interactions between various clinical factors and length of stay might be better understood through a thorough descriptive analysis before proceeding to predictive modeling. Third, our current analysis provides a solid foundation for future studies that could explore predictive modeling with expanded datasets and more sophisticated statistical approaches. We believe this stepwise approach allows for a more robust understanding of the fundamental relationships in our data.

Discussion:
- Please, expand more on the complications: There is exclusion/selection bias (due to excluding those with no follow up or incomplete data), single center, etc.

Reply:Thank you very much for your valuable comment.The changes have been made as requested.(page 19,Discussion part,paragraph4,line4-17)

Reviewer #2: The manuscript “Establishment and Validation of an Interactive Web-Based Calculator for Predicting Postoperative Functional Recovery in Metatarsal Fracture Patients: A LASSO Regression Model Approach” considers a relevant topic of interest for orthopedic and clinical researchers, particularly in the context of postoperative functional recovery in metatarsal fracture patients. The purposes of this study seem to be clear. However, there are some issues and concerns need to be addressed. Please see my comments below:
Abstract
1.The background section of the abstract is lacking some essential information. I recommend that the author provide additional relevant background details to strengthen the context of the study. Furthermore, there is an inconsistency in line spacing between the 'Objective' section and other sections of the manuscript. I suggest that the author adjust the line spacing to ensure uniformity throughout the document.

Reply:Thank you very much for your valuable comment.I apologize for my carelessness.I think the newly added background section effectively bridges the context(page 1,Abstract-Background,paragraph1)

Introduction
2. The introduction section lacks sufficient supporting literature, particularly in the area of clinical risk prediction models for metatarsal fractures. There is a lack of a comprehensive summary and analysis of previous studies, which makes it difficult to clearly identify the novel aspects of this research.

Reply:Thank you very much for your valuable comment.(page2-3,introduction part,paragraph1、2、3、5).After enrichment and modification, this study provides a systematic analysis and summary of the current research status of metatarsal fractures as follows:(1). Epidemiological characteristics:Metatarsal fractures account for 3.2-6.8% of all fractures, with an annual incidence of 67-75.4 per 100,000;Represents 88.5% of foot fractures, predominantly affecting young and middle-aged populations;Primarily caused by sports injuries and high-energy trauma

(2). Factors affecting fracture healing:Patient-related: age, gender, comorbidities, medication, and nutritional status;Non-patient-related: fracture characteristics, infection, internal fixation materials, etc;Vascular reconstruction plays a crucial role in fracture healing, while conditions such as diabetes may impede healing through microcirculatory effects

(3). Major complications:Metatarsalgia (most common), post-traumatic arthritis;Arch collapse, complex regional pain syndrome

(4). Limitations of previous studies:Lack of comprehensive epidemiological studies on postoperative functional recovery;Predominantly limited to specific populations or fracture types

(5).Research innovations:Implementation of LASSO regression for variable selection;Integration of interactive web calculator;Provision of personalized treatment decision support

Method
3.While the retrospective design of the study is clearly stated, it is recommended to discuss more about the limitations inherent in retrospective studies, such as selection bias and information bias. These potential biases should be addressed in the discussion section to explain how they might affect the interpretation of the results.

Reply:Thank you very much for your valuable comment.It has been added and explained in the discussion section.(page 19,Discussion part,paragraph 4 ,line 4-17).

4.The exclusion criteria are generally clear, but could be further refined. For example, the term "non-surgical treatment" needs more precise definition. What specific treatments are considered non-surgical? Additionally, for "old or pathological fractures," more detailed criteria should be provided to reduce ambiguity.

Reply:Thank you very much for your valuable comment.The changes have been made as requested.(page 3,Method-Patient selection part,line 8-21)

5.The data collection covers a wide range of factors, including preoperative, intraoperative, and postoperative variables. It would be helpful to present a table or checklist clearly listing the specific data collected. This would ensure that the level of detail is adequate for supporting subsequent analysis and conclusions.

Reply:Thank you very much for your valuable comment.The changes have been made as requested.(page 7,table 1,Classification of Potential Predictive Factors for Postoperative Functional Recovery in Metatarsal Fracture Patients)

6.While follow-up is primarily done by phone and medical record review, more details should be provided about the frequency and timing of follow-ups, as well as measures to ensure the accuracy of the follow-up data (e.g., how missing data will be handled). Telephone follow-ups may introduce information bias, and it is recommended to specify how missing data is addressed.

Reply:Thank you very much for your valuable comment.Since patients lost to follow-up were already excluded in the inclusion/exclusion process, they were not included in the study scope.

7.You mentioned the use of the AO/OTA classification system for fracture classification. It would be helpful to clarify how the imaging data was obtained and the standardization of imaging assessment. For instance, was the imaging data reviewed by a single radiologist or multiple assessors? If possible, mention whether automated tools were used to assist with classification.

Reply:Thank you very much for your valuable comment.The changes have been made as requested.(page 5,Data collection part,paragraph 3,line8-10)(The radiological data were evaluated by multiple study assessors, and no automated tools were used to assist in the classification process.)

8.In Figure 2, there is an inconsistency between the labels of panels A and B and their corresponding numbers. I strongly recommend improving the clarity of the figures and ensuring that the labeling is correct. Additionally, some data in the table show significant differences, but these differences have not been marked. Please check and correct the annotations accordingly.

Reply:Thank you very much for your valuable comment.The labels for panels A and B were verified to be correctly matched with their corresponding figures.

Discussion& Conclusion
9.The Discussion and Conclusion sections provide a comprehensive interpretation of the study results. However, the manuscript contains numerous formatting errors throughout. I strongly recommend that the authors carefully review and correct these formatting issues.

Reply:Thank you very much for your valuable comment.I apologize for my carelessness.The changes have been made as requested.

Yours sincerely,

* Corresponding author:

Hongzhi Lv.E-mail address: [190099199@qq.com](mailto:190099199@qq.com)

Yingze Zhang.E-mail address:[dryzzhang@126.com](mailto:dryzzhang@126.com)

Wei Chen.E-mail address:surgeonchenwei@126.com
