# Supplementary material for: Establishment and validation of an interactive web-based calculator for predicting postoperative functional recovery in metatarsal fracture patients: A LASSO regression model approach
Source: PLoS One. 2025 Jun 3;20(6):e0323609. doi: 10.1371/journal.pone.0323609 (PMC12132955; doi:10.1371/journal.pone.0323609)
Supplement: Attachment — Submitted filename: Reviewers Comments.docx [file pone.0323609.s003.docx]

**Reviewer Comments:**

This study developed and validated an interactive web-based calculator based on LASSO regression to predict the postoperative functional recovery of metatarsal fracture patients, and evaluated its predictive performance and clinical applicability through internal and external validation. However, there are several issues affecting the overall quality of the paper, as follows: The Abstract of this paper is too long, exceeding the recommended word count. The Introduction not only fails to deeply explore the current situation of clinical prediction of postoperative functional recovery, but merely lists basic epidemiological data. Moreover, there are few references. It is recommended to add more references in related fields to make this research well-founded. The research design has limitations. The single-center retrospective study design, sample selection, and exclusion criteria lead to selection bias, and patients receiving conservative treatment are overlooked. There are also flaws in data collection and analysis, which are prone to information bias and lack of multicollinearity testing. The Discussion section does not conduct an in-depth comparative analysis of the results, fails to analyze the reasons for differences and the mechanisms of special phenomena, and lacks consideration of how to improve the limitations when elaborating on them. Thus, it provides limited guidance for follow-up research.
