# Supplementary material for: Establishment and validation of an interactive web-based calculator for predicting postoperative functional recovery in metatarsal fracture patients: A LASSO regression model approach
Source: PLoS One. 2025 Jun 3;20(6):e0323609. doi: 10.1371/journal.pone.0323609 (PMC12132955; doi:10.1371/journal.pone.0323609)
Supplement: Attachment — Submitted filename: Response_to_Reviewers_auresp_2.doc [file pone.0323609.s004.doc]

Responses to Questions(Major Revision)

Dear Editors and Reviewers:

We greatly appreciate your continued attention and the additional comments provided on our manuscript. Thank you for taking the time to review our revised submission and offering further suggestions for improvement.

We are grateful for your thorough second review, which has helped us identify areas that still needed refinement. Your persistent dedication to improving the quality of our work is truly valuable, and we have carefully addressed each of your new comments with detailed revisions.

The additional feedback has allowed us to further enhance the clarity and rigor of our manuscript. We have diligently worked to incorporate all your latest suggestions into this second revision, aiming to meet the high standards of the journal.

We sincerely hope that the current version addresses all your concerns satisfactorily. If there are any remaining issues that require attention, we welcome your further guidance and feedback.

Thank you again for your continued support and commitment to helping us improve our work!

Reviewer Comments:

This study developed and validated an interactive web-based calculator based on LASSO regression to predict the postoperative functional recovery of metatarsal fracture patients, and evaluated its predictive performance and clinical applicability through internal and external validation. However, there are several issues affecting the overall quality of the paper, as follows:

1. The Abstract of this paper is too long, exceeding the recommended word count.

Reply:Thank you very much for your valuable comment.The abstract has been condensed.(page 1-2, part Abstract )

2. The Introduction not only fails to deeply explore the current situation of clinical prediction of postoperative functional recovery, but merely lists basic epidemiological data.

Reply:Thank you very much for your valuable comment.After careful consideration and verification, I added some language to discuss the current state of clinical prediction for postoperative functional recovery and supplemented it with relevant epidemiological data.(page2 , part Introduction ,line36-41.)

3. Moreover, there are few references. It is recommended to add more references in related fields to make this research well-founded.

Reply:Thank you very much for your valuable comment.I have added references in multiple aspects, including the application and advantages of the LASSO method (page 23, part Reference, 24-28), web page generation and front-end UI development, as well as methods for front-end and back-end connection (page 23, part Reference, 40-43), and enrichment of related research fields in the discussion section (pages 25-26, part Reference, 50-52, 55, 56, 62, 72, 73).

4.The research design has limitations. The single-center retrospective study design, sample selection, and exclusion criteria lead to selection bias, and patients receiving conservative treatment are overlooked. There are also flaws in data collection and analysis, which are prone to information bias and lack of multicollinearity testing.

Reply:Thank you very much for your valuable comment.I have supplemented the discussion section with the difficult-to-overcome problems in this study, added limitations, and provided many insights for future research directions based on the study's limitations.(page21, part Discussion ,line26-40.)

5. The Discussion section does not conduct an in-depth comparative analysis of the results, fails to analyze the reasons for differences and the mechanisms of special phenomena, and lacks consideration of how to improve the limitations when elaborating on them. Thus, it provides limited guidance for follow-up research.

Reply:Thank you very much for your valuable comment.In the discussion section, I enriched the original content in the exploration of each key variable, and provided reasonable explanations for special research findings with supporting literature. For example, the complex dose-dependent effects of nicotine, and how the different impacts of hilly and mountainous terrain support the "optimal position" hypothesis (page 20, part Discussion, lines 5-24, 34-39; page 21, lines 6-10). At the end of the discussion, I added how to improve the limitations and what guidance this provides for subsequent research (page 21, part Discussion, lines 26-40).

Review Comments to the Author

Reviewer #2: The author has addressed most of my previous comments, and I appreciate the revisions made in the manuscript. However, upon reviewing the revised version, I still have some concerns and questions that need to be addressed. Please find my detailed comments below.

1. The reviewers regarded that the author's description of the Losso algorithm in the methods section was too simplistic. At the same time, there is a lack of visual expression of the overall process of the research, and it may be difficult to clearly present the complete process of the research with a simple text description.

Reply:Thank you very much for your valuable comment.I have added the advantages of using the LASSO algorithm in the introduction section (page 3, part Introduction, lines 1-14), and created a research methodology flowchart in the methods section to clearly present the complete research process (page 6, part Method, fig 1).

2. When the authors included the data, the dataset included both male and female gender characteristics, but the authors did not make a specific analysis of the predictions for these two genders in the results and discussions. In addition, there were differences in the characteristics used by the authors between the two genders, such as whether some women did not smoke and whether this affected the accuracy of the algorithm.

Reply:Thank you very much for your valuable comment.Indeed, this is a very good suggestion. The reason for not separating the studies at the time was to ensure the universality of the final model, to obtain generalized results, and to make the model more convenient and easier to interpret. Regarding this, I have explained the drawbacks in the discussion section, and I hope these limitations can provide good directions for future research.

3. The author has developed a web-based calculator in his research, but the author does not give a detailed description of the development of the front-end UI part of the web and the methods of front-end and back-end connection in the methods section.

Reply:Thank you very much for your valuable comment.I have modified and enriched the web development section, which includes the development of the Web front-end UI and methods for connecting the front-end and back-end. The changes have been made as requested (page 5, part Method, lines 31-41).

4. The current study has used machine learning methods to predict sports injuries with relatively accurate results Accurately and effectively predict the ACL force: Utilizing biomechanical landing pattern before and after-fatigue (https://doi.org/10.1016/j.cmpb.2023.107761) The authors may consider exploring the limitations and future research in depth by incorporating the latest relevant research to inform the reader's thinking.

Reply:Thank you very much for your valuable comment.I have adopted your valuable suggestions by adding more in-depth supplements to the discussion of relevant impact factors and identifying future research directions worth exploring while examining the limitations in depth.(Reference 39)、(page 20, part Discussion ,line5-24,34-39；page 21, line6-10.)、(page21, part Discussion ,line26-40.).

5. Upon review, I still have concerns regarding the quality of the presentation of the key data visualizations in the manuscript. Additionally, the author must take further steps to ensure that the entire manuscript is free of grammatical issues in the next revision.

Reply:Thank you very much for your valuable comment.I apologize for any inconvenience caused. I have rechecked the article and confirmed that there are no grammatical issues.

Yours sincerely,

* Corresponding author:

Hongzhi Lv.E-mail address: [190099199@qq.com](mailto:190099199@qq.com)

Yingze Zhang.E-mail address:[dryzzhang@126.com](mailto:dryzzhang@126.com)

Wei Chen.E-mail address:surgeonchenwei@126.com
