# Supplementary material for: Establishment and validation of an interactive web-based calculator for predicting postoperative functional recovery in metatarsal fracture patients: A LASSO regression model approach
Source: PLoS One. 2025 Jun 3;20(6):e0323609. doi: 10.1371/journal.pone.0323609 (PMC12132955; doi:10.1371/journal.pone.0323609)
Supplement: Attachment — Submitted filename: Response_to_Reviewers_auresp_3.doc [file pone.0323609.s005.doc]

Responses to Questions(Major Revision)

Dear Editors and Reviewers:

Thank you very much for your continued interest in our paper and your valuable suggestions. We have completed the third round of revisions based on your comments, and we hope that the current manuscript can better present our research results. All the changes and markings have been made using the revision model in the attachment. If there are still issues that need to be further addressed, please feel free to remind us and we will do our best to cooperate and improve.

Once again, we sincerely thank you for your guidance and help, we look forward to your positive feedback, and sincerely hope that the revised manuscript will meet the publication standards.

Journal Requirements:
Please review your reference list to ensure that it is complete and correct. If you have cited papers that have been retracted, please include the rationale for doing so in the manuscript text, or remove these references and replace them with relevant current references. Any changes to the reference list should be mentioned in the rebuttal letter that accompanies your revised manuscript. If you need to cite a retracted article, indicate the article’s retracted status in the References list and also include a citation and full reference for the retraction notice.

Reply:Thank you very much for your valuable comment.We have reviewed the entire reference list and confirmed that no retracted papers were cited.Minor corrections to typographical errors in the reference list have been made(page 22-26, part Reference).

Yours sincerely,

* Corresponding author:

Hongzhi Lv.E-mail address: [190099199@qq.com](mailto:190099199@qq.com)

Yingze Zhang.E-mail address:[dryzzhang@126.com](mailto:dryzzhang@126.com)

Wei Chen.E-mail address:surgeonchenwei@126.com
